# Molecular mechanism of muscarinic acetylcholine receptor M3 interaction with Gq

**DOI:** 10.1038/s42003-024-06056-1

**Published:** 2024-03-23

**Authors:** Donghee Ham, Asuka Inoue, Jun Xu, Yang Du, Ka Young Chung

**Affiliations:** 1 School of Pharmacy, Sungkyunkwan University, 2066 Seobu-ro, Jangan-gu, Suwon 16419 Republic of Korea; 2 Graduate School of Pharmaceutical Sciences, Tohoku University, 6-3, Aoba, Aramaki, Aoba-ku, Sendai, Miyagi 980-8578 Japan; 3 Molecular and Cellular Physiology, School of Medicine, Stanford University, Stanford, CA 94305 USA; 4 Kobilka Institute of Innovative Drug Discovery, School of Medicine, Shenzhen Futian Biomedical Innovation R&D Center, the Chinese University of Hong Kong, Shenzhen, 518172 Guangdong China

**Keywords:** Biophysical chemistry, Hormone receptors

## Abstract

Muscarinic acetylcholine receptor M3 (M3) and its downstream effector Gq/11 are critical drug development targets due to their involvement in physiopathological processes. Although the structure of the M3-miniGq complex was recently published, the lack of information on the intracellular loop 3 (ICL3) of M3 and extensive modification of Gαq impedes the elucidation of the molecular mechanism of M3-Gq coupling under more physiological condition. Here, we describe the molecular mechanism underlying the dynamic interactions between full-length wild-type M3 and Gq using hydrogen-deuterium exchange mass spectrometry and NanoLuc Binary Technology-based cell systems. We propose a detailed analysis of M3-Gq coupling through examination of previously well-defined binding interfaces and neglected regions. Our findings suggest potential binding interfaces between M3 and Gq in pre-assembled and functionally active complexes. Furthermore, M3 ICL3 negatively affected M3-Gq coupling, and the Gαq AHD underwent unique conformational changes during M3-Gq coupling.

## Abstract (web summary)

Analysis of M3-Gq coupling utilized fulllength proteins and techniques like HDX-MS & NanoBiT assay, revealing potential binding interfaces in the preassembled and agonistactive complexes and critical regions for the complex formation and Gq activation.

## Introduction

Muscarinic acetylcholine receptors (mAChRs) are G protein-coupled receptors (GPCRs) with five subtypes, M1 to M5^1–3^. mAChRs are involved in several pathological conditions, such as central nervous system diseases, overactive bladder, chronic obstructive pulmonary disease, and Sjögren’s syndrome^4^. Therefore, developing mAChR-targeting medicines^5–7^ and understanding the molecular mechanisms of mAChR signaling are of great interest^8–12^.

Vertebrate GPCRs are categorized into five classes, rhodopsin (class A), secretin (class B1), adhesion (class B2), glutamate (class C), and frizzled/taste2 (classes F and T)^13^; mAchRs are classified as rhodopsin-like (class A) receptors. As the name implies, GPCRs act via coupling with G proteins, which are composed of three subunits, Gα, Gβ, and Gγ^14^. In the inactive state, Gα is occupied by guanosine diphosphate (GDP) and forms a heterotrimer with Gβγ^15^. Activated GPCRs interact with and induce conformational changes in G proteins, resulting in GDP release^16^. Due to an approximately 10-fold higher cellular concentration of guanosine triphosphate (GTP) than GDP^17^, the empty Gα is quickly occupied by GTP, resulting in the activation and dissociation of Gα from the receptor and Gβγ^18^. The GTPase activity within Gα hydrolyzes GTP into GDP, leading to inactive Gαβγ heterotrimer formation. Based on function and sequence similarity, Gα is categorized into four subfamilies—Gαs, Gαi/o, Gαq/11, and Gα12/13^14,15^. The odd-numbered mAChRs, M1, M3, and M5 primarily couple to Gαq/11 subfamilies while M2 and M4 primarily couple to Gαi/o subfamilies^19^.

**Fig. 1:**
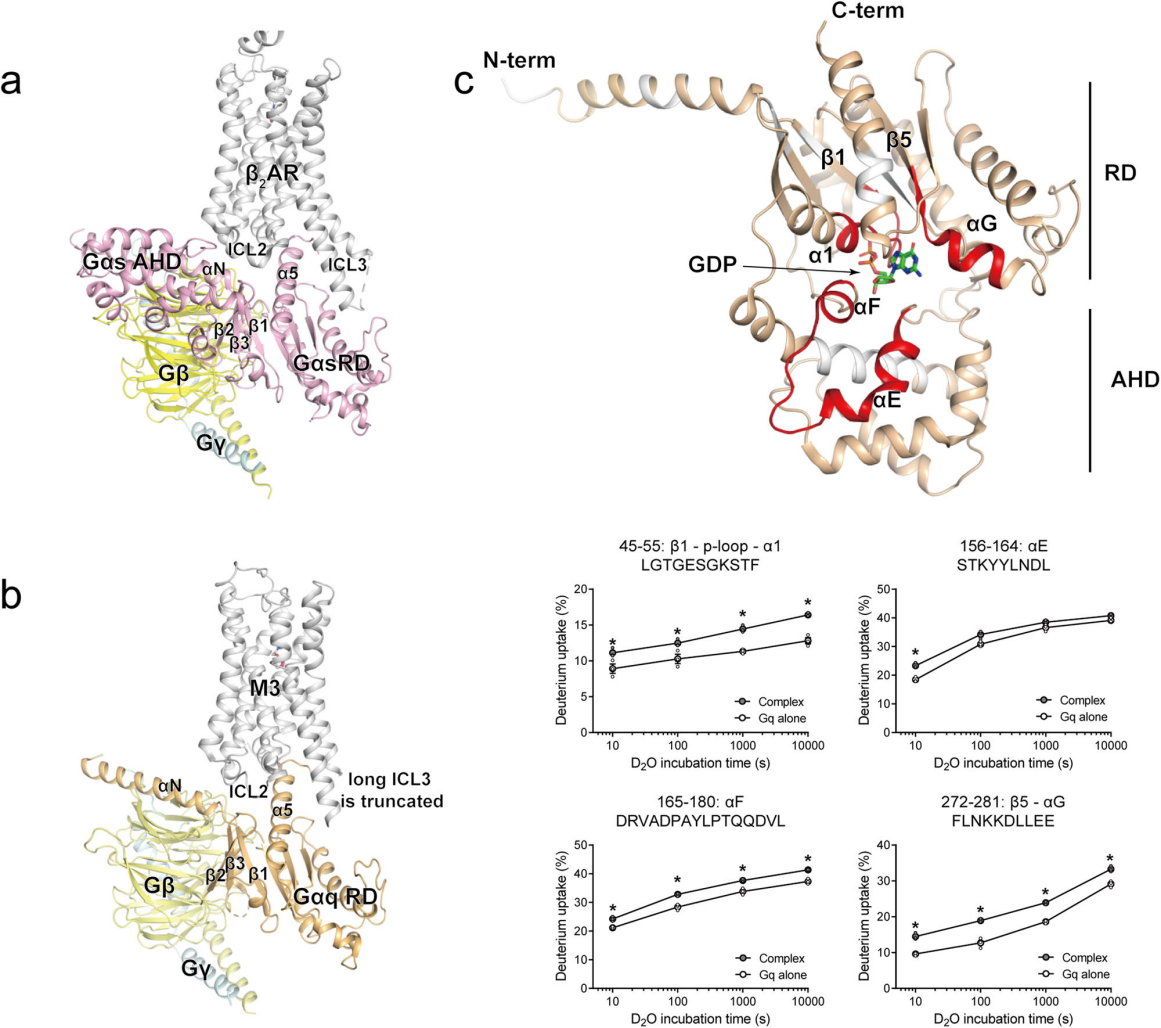
The structure of the GPCR-G protein complex and HDX profiles of Gαq near the nucleotide-binding pocket upon co-incubation with M3. **a**, **b** The structure of β_2_AR–heterotrimeric Gs (PDB: 3SN6) (**a**) and M3–heterotrimeric Gq (PDB: 8E9Z) (**b**). β_2_AR and M3 are colored grey; whereas Gαs, Gαq, Gβ, and Gγ are colored light pink, light orange, yellow, and light cyan, respectively. **c** Changes in the HDX profile near the nucleotide-binding pocket of Gαq upon co-incubation of Gq with agonist-bound M3. Regions with increased HDX near the nucleotide-binding pocket are colored red on the crystal structure of GDP- and YM-254890-bound inactive Gαq (PDB: 3AH8), and the deuterium uptake plots of selected peptides are provided as graphs. The regions in which peptides were not identified are shown in white. Results were derived from three independent experiments and the statistical significance of differences was determined using Student’s t-test (*p < 0.05). Data are presented as mean ± standard error of the mean. Smaller symbols are individual data points.

The first GPCR-G protein complex structure revealed was the β_2_-adrenergic receptor (β_2_AR)-Gs complex (Fig. 1a), and many other GPCR-G protein complexes in various coupling pairs have since been identified (450 GPCR-G protein complex structures and 122 unique GPCR-G protein complexes according to GPCRdb.org)^13^. These structures revealed the receptor-G protein binding interfaces and allosteric conformational changes upon complex formation^20,21^. The intracellular side of GPCRs, including the intracellular loops (ICLs), plays an important role in G protein interactions. Specifically, ICL2 and ICL3 have been suggested to be the major binding sites for G proteins^22–25^ (Fig. 1a).

Interestingly, the length of ICL3 in class A GPCRs varies from 2 to 211 residues. Although most class A receptors (approximately 75% of class A GPCRs, except olfactory receptors) contain < 10 residues in ICL3, a small portion of class A receptors (approximately 5% of class A GPCRs, except olfactory receptors) contain >100 residues (Supplementary Fig. 1). Moreover, mAChRs have extremely long ICL3s, and according to GPCRdb.org, M1, M2, M3, M4, and M5 have 128, 152, 211, 156, and 200 residues in ICL3, respectively. Notably, M3 has the longest ICL3 among class A receptors^13^. Among the mAChRs, the structures of M1-G11, M2-Go, M3-Gq (Fig. 1b), and M4-Go complexes have been revealed^11,12,26,27^. However, the receptors in these structures are extensively modified to form stable complexes (Supplementary Table 1); they are often truncated at flexible regions, such as the ICL3, N-terminus, and/or C-tail, and/or mutated to generate designer receptors exclusively activated by designer drugs (DREADDs). Therefore, these structures do not provide a potential role for ICL3 in the mAChR interaction with G proteins.

All currently available GPCR-Gq complex structures contain extensively modified Gαq to form stable complexes (Supplementary Table 1). Gαq is modified as a chimeric Gα and is often engineered as miniGαq, which is primarily derived from miniGαs with the α-helical domain (AHD) truncated (compare Gα in Fig. 1a, b) and a few selected regions mutated. Additionally, Gq in these complex structures is bound to auxiliary proteins, such as scFv16. In some structures, the receptor and Gq were forced into stable complexes using HiBiT, a high-affinity version of the NanoLuc Binary Technology (NanoBiT) (Supplementary Table 1). Therefore, the reported GPCR-Gq complex structures, including the M3-miniGq complex, may differ from those of GPCR-Gq complexes under physiological conditions. Here, we studied the molecular mechanisms underlying the interactions between M3 and Gq using full-length wild-type (WT) M3 and Gq to understand their interactions without modifications. We used a combination of techniques, including hydrogen/deuterium exchange mass spectrometry (HDX-MS) and NanoBiT-based cell systems, to elucidate the conformational changes in M3 and Gq upon complex formation and the roles of specific regions in their coupling.

## Results

### Functional complex formation between the purified full-length WT M3 and Gq in vitro

We first examined if the purified full-length WT M3 and un-modified Gq can functionally couple by performing the BODIPY-FL-GTPγS fluorescence assay. The BODIPY fluorescence is low in the solvent but increases when BODIPY-FL-GTPγS is inserted into Gα^28,29^. When the purified full-length WT M3 was added to Gq, the BODIPY fluorescence increased (Supplementary Fig. 2), which suggests that M3 successfully induced GDP release from Gq implying functional coupling of the purified full-length WT M3 and un-modified Gq.

### Conformational changes near the Gαq nucleotide-binding pocket upon co-incubation with M3

To make stable M3-Gq complexes for structural analysis, we co-incubated purified full-length WT M3 and unmodified Gq followed by apyrase treatment; apyrase was used to remove the released GDP and prevent re-binding of GDP to the complex^30^. This GDP-free receptor-bound state is the intermediate state of G proteins before fully activated by binding of GTP and has been used to analyze the high-resolution structures of the GPCR-G protein complexes. Unfortunately, however, we could not obtain a stable M3-Gq complex, which suggests that the functional complex formed by the purified full-length WT M3 and un-modified Gq is very weak. Due to the weak nature of the M3-Gq complex, a previous structural analysis used HiBiT technology to form the stable complex^26^. Although we could not obtain the stable complex, we pursued the conformational analysis using HDX-MS.

HDX-MS measures the exchange between amide hydrogen atoms in a protein and deuterium in the solvent, revealing the conformational dynamics of the protein^31,32^. HDX-MS has been successfully used to analyze dynamic conformational changes during GPCR-G protein coupling, such as β_2_AR-Gs coupling and M2-Gi/o coupling^33–36^. These studies suggest the conformational changes at the GPCR-G protein-binding interfaces and allosteric conformational changes induced by GPCR-G protein interaction, GDP release, or GTPγS binding. Moreover, HDX-MS has been often used to probe binding interfaces or conformational changes induced by even very weak interactions between two proteins^34,37^. Thus, one of the advantages of HDX-MS is that proteins can be analyzed without extensive modifications, such as truncation of flexible regions or introduction of stabilizing mutations. Therefore, HDX-MS is a suitable tool for studying the dynamic conformational changes during the relatively weak interaction between full-length WT M3 and unmodified Gq.

Apyrase itself did not affect the conformational dynamics of Gq as we did not detect differences in HDX levels between Gq with and without apyrase treatment (Supplementary Data 1).

When the HDX-MS profiles of Gq were analyzed with or without co-incubation with M3 (Supplementary Data 2), the regions near the nucleotide-binding pocket showed higher HDX levels in the Gq co-incubated with M3 than in the Gq alone (Fig. 1c). The Gα subunit is composed of a Ras-like GTPase domain (RD) and an AHD between which GDP or GTP is located (Fig. 1c). In the GDP-released state, AHD moves away, exposing the nucleotide-binding pocket (Supplementary Fig. 3a). Therefore, the higher HDX levels near the nucleotide-binding pocket imply that these regions were exposed to the buffer and/or became more dynamic potentially due to M3-induced GDP release.

### Increased conformational dynamics in the Gαq AHD upon co-incubation with M3

**Fig. 2:**
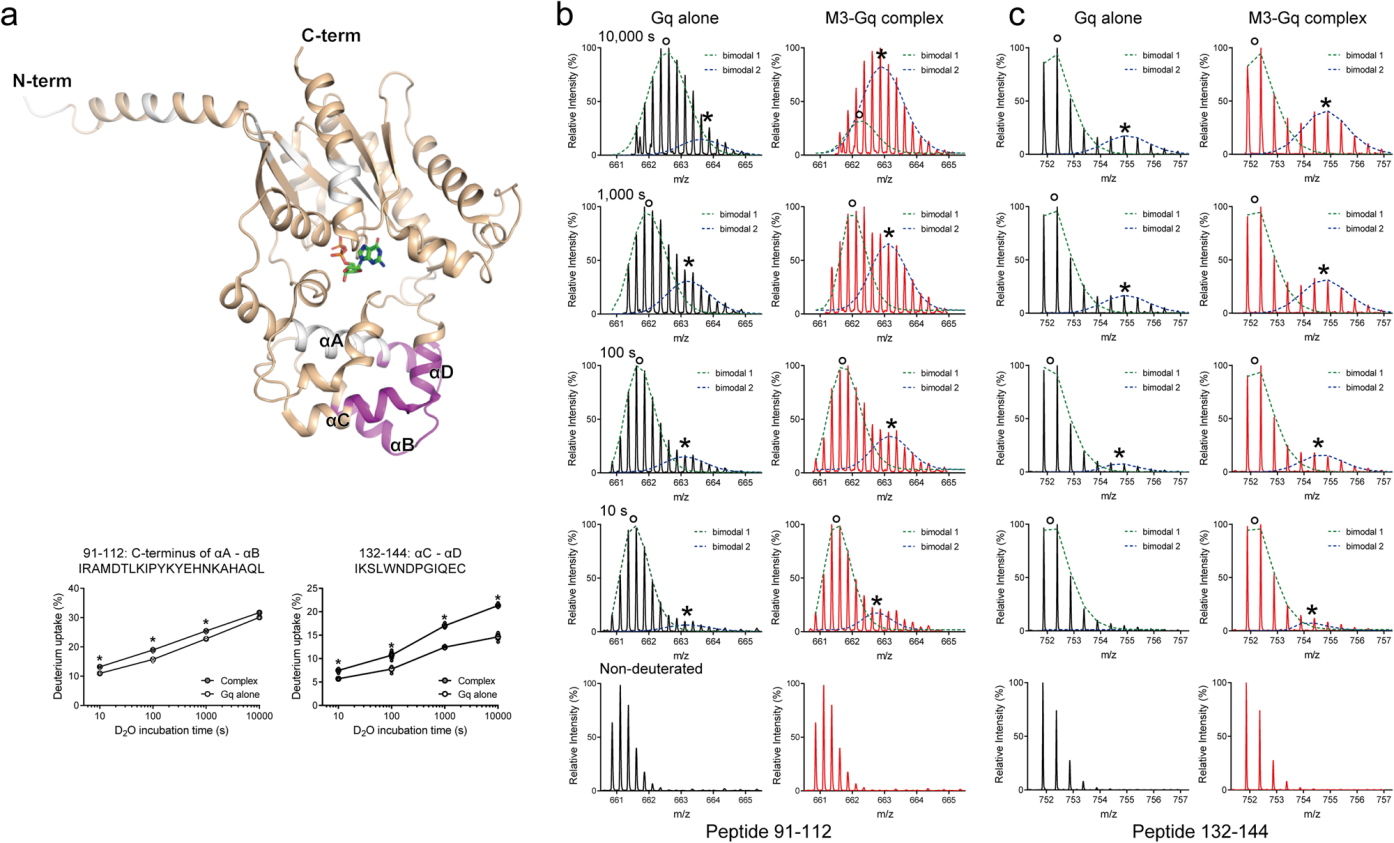
EX1 kinetic profile of the Gαq AHD upon co-incubation with M3. **a** Regions with EX1 kinetics are colored purple on the structure of Gαq (PDB: 3AH8). The deuterium uptake plots of peptides from purple-colored regions of the Gαq AHD are provided as graphs. The regions in which peptides were not identified are shown in white. Results were derived from three independent experiments and the statistical significance of differences was determined using Student’s t-test (*p < 0.05). Data are presented as mean ± standard error of the mean. Smaller symbols are individual data points. **b**, **c** Raw mass spectra from various deuterated time points (non-deuterated, 10 s, 100 s, 1000 s, and 10,000 s) of peptides 91–112 (**b**) and 132–144 (**c**). The bimodal spectral envelopes were deconvolved using HX-Express3 (https://www.hxms.com/HXExpress/)^67^.

The HDX-MS profiles revealed unique HDX kinetics within two regions of the Gαq AHD (Fig. 2). A protein’s folding status can be examined by carefully inspecting its HDX mass spectrum. Proteins in solution undergo constant local unfolding and refolding, and amide hydrogen atoms can be switched for deuterium in the buffer when the protein is in the unfolded state^38,39^. In most cases, the HDX rate is much slower than the unfolding/refolding rate, and thus the HDX mass spectra show a gradual increase in the average mass (binomial isotropic distribution), known as EX2 kinetics^39^. In rare cases, the unfolding/refolding rate is much slower than the HDX rate, and HDX occurs cooperatively in a single unfolding event prior to refolding^40–43^. Consequently, the mass spectra show two distinct mass envelopes (a bimodal isotropic distribution), called EX1 kinetics. With pure EX1 kinetics, the low- and high-mass envelopes do not change their position (m/z) as a function of the exchange duration; however, the peak intensity of the low-mass envelope decreases, whereas that of the high-mass envelope increases. The existence of EX1 kinetics suggests conformational heterogeneity, implying that proteins exist as an ensemble of folded and unfolded states^44^.

We observed mixed EX1/EX2 kinetics in two regions of the Gαq AHD (Fig. 2). Most peptides from Gαq showed a binomial isotropic distribution; however, two closely located peptides (Fig. 2a; peptides 91–112 and 132–144) showed a bimodal isotropic distribution (Fig. 2b, c). In this bimodal distribution, the low-mass distribution (marked as ◦) corresponds to a slower-exchanging conformer, and the high-mass distribution (marked as *) corresponds to a faster-exchanging conformer (Fig. 2b, c). The slower-exchanging conformers are relatively folded (buried or ordered), whereas the faster-exchanging conformers are relatively unfolded (exposed or flexible).

In our data, because both the intensity of the faster-exchanging conformer and the m/z of the slower-exchanging conformer increased with increasing deuterium exposure, the exchange did not occur exclusively through an EX1 mechanism, but instead involved a mixture of EX1/EX2 kinetics. The proportion of faster-exchanging conformers was higher in the Gq co-incubated with M3 than in the Gq alone (Fig. 2b, c); therefore, the overall deuterium uptake levels were higher with these peptides in the Gq co-incubated with M3 than in the Gq alone (Fig. 2a). Thus, the purple regions in Fig. 2a undergo increased conformational dynamics (potentially partial unfolding of the-helices) when Gq forms a complex with M3, and GDP is released. These regions are located far from the M3 binding interfaces and GDP-binding sites; therefore, increased conformational dynamics in these regions may occur through allosteric conformational changes transmitted from the GDP-binding site.

### The C-terminus of Gαq α5 forms shallow or unstable interactions with M3 but remains critical for M3-Gq coupling

To understand the binding interface, we sought the regions that showed lower HDX in the Gq co-incubated with M3 than in the Gq alone because the binding interfaces become less exposed to the buffer and/or less dynamic in the complex than in the Gq alone. In the published cryo-electron microscopy (cryo-EM) structure of the M3-miniGq complex (Fig. 1b), the overall receptor-G protein interfaces are similar to other class A GPCR-G protein-binding interfaces (Fig. 1a), with the C-terminus of Gαq α5 extensively interacting with the M3 cytosolic core.

**Fig. 3:**
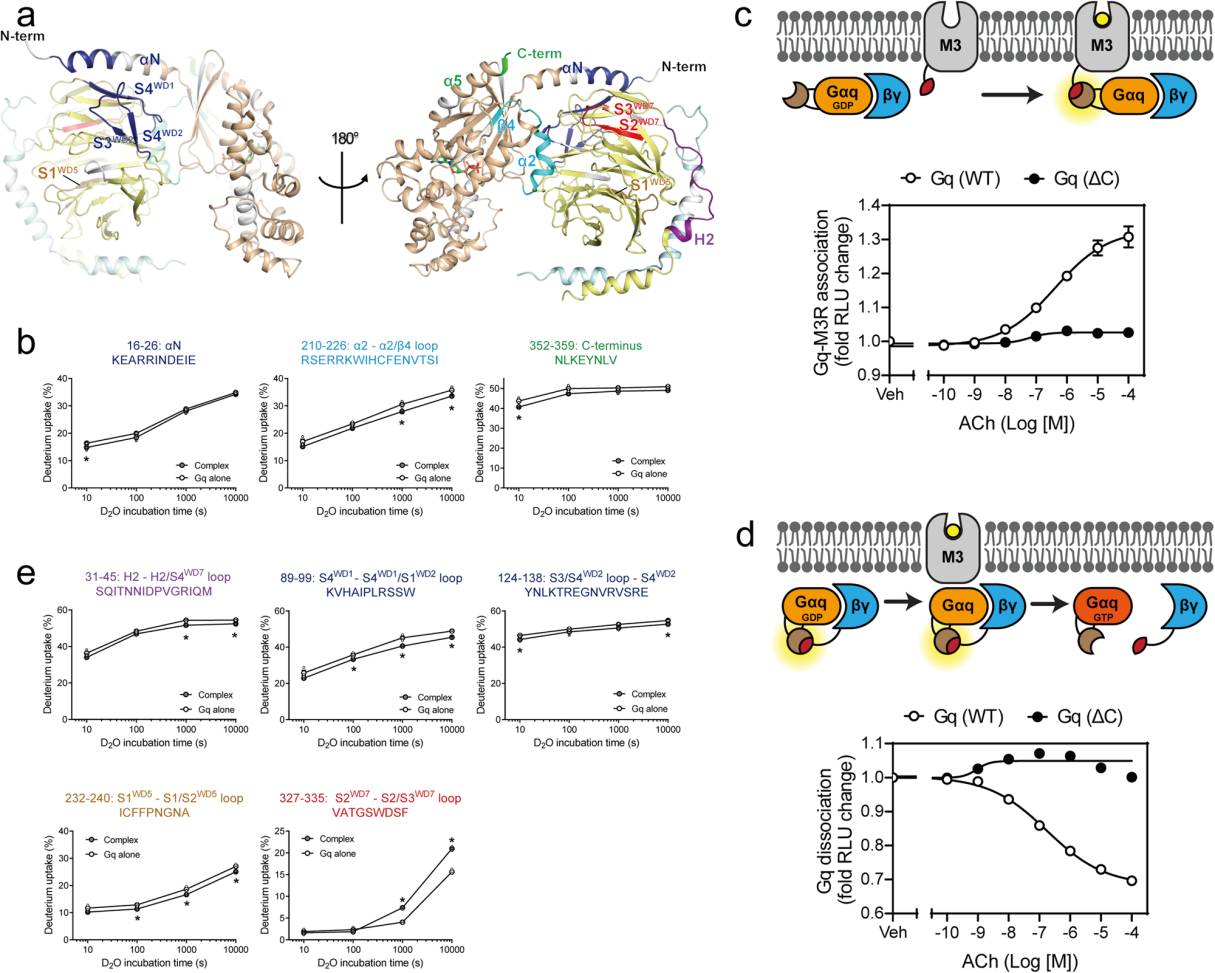
Changes in HDX profiles of heterotrimeric Gq upon co-incubation with M3 and the role of Gαq α5 on M3-Gq coupling. **a**, **b**, **e** Changes in the HDX profiles of Gαq and Gβ_1_ upon co-incubation of Gq with agonist-bound M3. Regions with altered HDX profiles are color-coded on the structure of inactive heterotrimeric Gq (PDB: 3AH8) (**a**), and the deuterium uptake plots of selected peptides from the color-coded regions of Gαq (**b**) and Gβ_1_ (**e**) are provided as graphs. The regions in which peptides were not identified are shown in white. Results were derived from three independent experiments and the statistical significance of differences was determined using Student’s *t*-test (*p < 0.05). Data are presented as mean ± standard error of the mean. Smaller symbols are individual data points. **c** Schematic representation and quantification of the NanoBiT-G-protein interaction assay. M3–SmBiT was expressed together with the wild-type (WT) or C-terminally truncated (∆C) Gαq–LgBiT, Gβ_1_, and Gγ_2_ constructs. Symbols and error bars represent mean and SEM, respectively, from three independent experiments with each performed in duplicate. Note that, in many data points, error bars are smaller than the size of the symbols. **d** Schematic representation and quantification of the-NanoBiT-G-protein dissociation assay. M3, Gαq–LgBiT, SmBiT–Gγ_2_, and Gβ_1_ were co-expressed. Symbols and error bars represent mean and SEM, respectively, from three independent experiments with each performed in duplicate. Note that, in many data points, error bars are smaller than the size of the symbols.

As expected, the C-terminus of Gαq α5 showed lower HDX in the Gq co-incubated with M3 than in the Gq alone (Fig. 3a, green-colored region, and Fig. 3b, peptide 352–359). However, the lower HDX level in the Gq co-incubated with M3 (3% lower than the Gq alone) was much smaller than that in other GPCR-G protein complexes (i.e., approximately 40% in β_2_AR-Gs, 20% in the A_2_A-Gs complex, and 30% in the M2-Gi/o complex)^33–35^. This suggests that the engagement of the C-terminus of Gq α5 with the M3 cytosolic core may be shallower or more unstable than that of β_2_AR-Gs, A_2_A-Gs, or M2-Gi/o complexes. The shallow or unstable nature of this interaction may explain the unstable M3-Gq complex and the need for HiBiT-assisted complex formation^26^.

The C-terminus of Gα α5 has been generally considered a critical region for interaction with receptors and selective coupling between a receptor and G protein^45–50^. As we observed relatively small HDX changes at the C-terminus of Gαq α5 compared to those in other GPCR-G protein complexes, we investigated whether the interaction at the C-terminus of Gαq α5 is important for M3-Gq coupling. Consequently, we compared WT and C-terminal-truncated Gαq using previously established NanoBiT-based cell systems to measure the M3-Gq interaction and M3-mediated Gq activation (i.e., GTP-binding-triggered Gβγ dissociation from Gα)^50,51^. To measure the interaction between Gq and M3, we used the Gαq construct harboring the NanoBiT large fragment (LgBiT) in the Gαq AHD and the M3 construct containing the small fragment (SmBiT) at the C-terminus (Fig. 3c). To monitor the M3-mediated Gq activation, we used the LgBiT-fused Gαq and the Gγ_2_ containing SmBiT at the N-terminus (Fig. 3d). When the last five residues at the C-terminus of Gαq α5 (i.e., EYNLV) were truncated, both the interaction with M3 and the M3-induced activation were almost completely abolished (Fig. 3c, d). Therefore, during M3-Gq coupling, the C-terminus of Gαq α5 forms a shallow or relatively unstable interaction with M3; however, this interaction remains critical for the efficient M3-Gq coupling.

### HDX-MS suggests unique potential binding interfaces between M3 and Gq

Interestingly, other regions of Gq that did not form contacts with M3 in the published cryo-EM structure of the M3-miniGq complex showed altered HDX levels in the Gq co-incubated with M3 compared with those in the Gq alone (Supplementary Data 2). The N-terminal region of Gαq αN (Fig. 3a, blue-colored region, and Fig. 3b, peptide 16–26) and its neighboring regions in Gβ_1_ (Fig. 3a, blue-colored regions, and Fig. 3e, peptides 89–99 and 124–138) showed lower HDX levels in the Gq co-incubated with M3 than in the Gq alone, suggesting that these regions become less exposed to the buffer and/or are conformationally more rigid in the complex than in the Gq alone. Gαq α2 through the α2/β4 loop (Fig. 3a, cyan-colored region, and Fig. 3b, peptide 210–226), Gβ_1_ H2 through S4^WD7^ (Fig. 3a, violet-colored region, and Fig. 3e, peptide 31–45), and Gβ_1_ S1^WD5^ through S1^WD5^/S2^WD5^ loop (Fig. 3a, brown-colored region, and Fig. 3e, peptide 232–240) also showed lower HDX levels in the Gq co-incubated with M3 than in the Gq alone, suggesting that these regions become less exposed to the buffer and/or are conformationally more rigid in the complex than in the Gq alone. Within Gβ_1_, S2^WD7^ through S2^WD7^/S3^WD7^ loop showed higher HDX levels in the Gq co-incubated with M3 than in the Gq alone (Fig. 3a, red-colored region, and Fig. 3e, peptide 327–335), suggesting that this region becomes conformationally more dynamic in the complex than in the Gq alone. These results suggest that the blue, cyan, violet, and brown-colored regions in Fig. 3a may be the binding interfaces with M3, which are further discussed below.

**Fig. 4:**
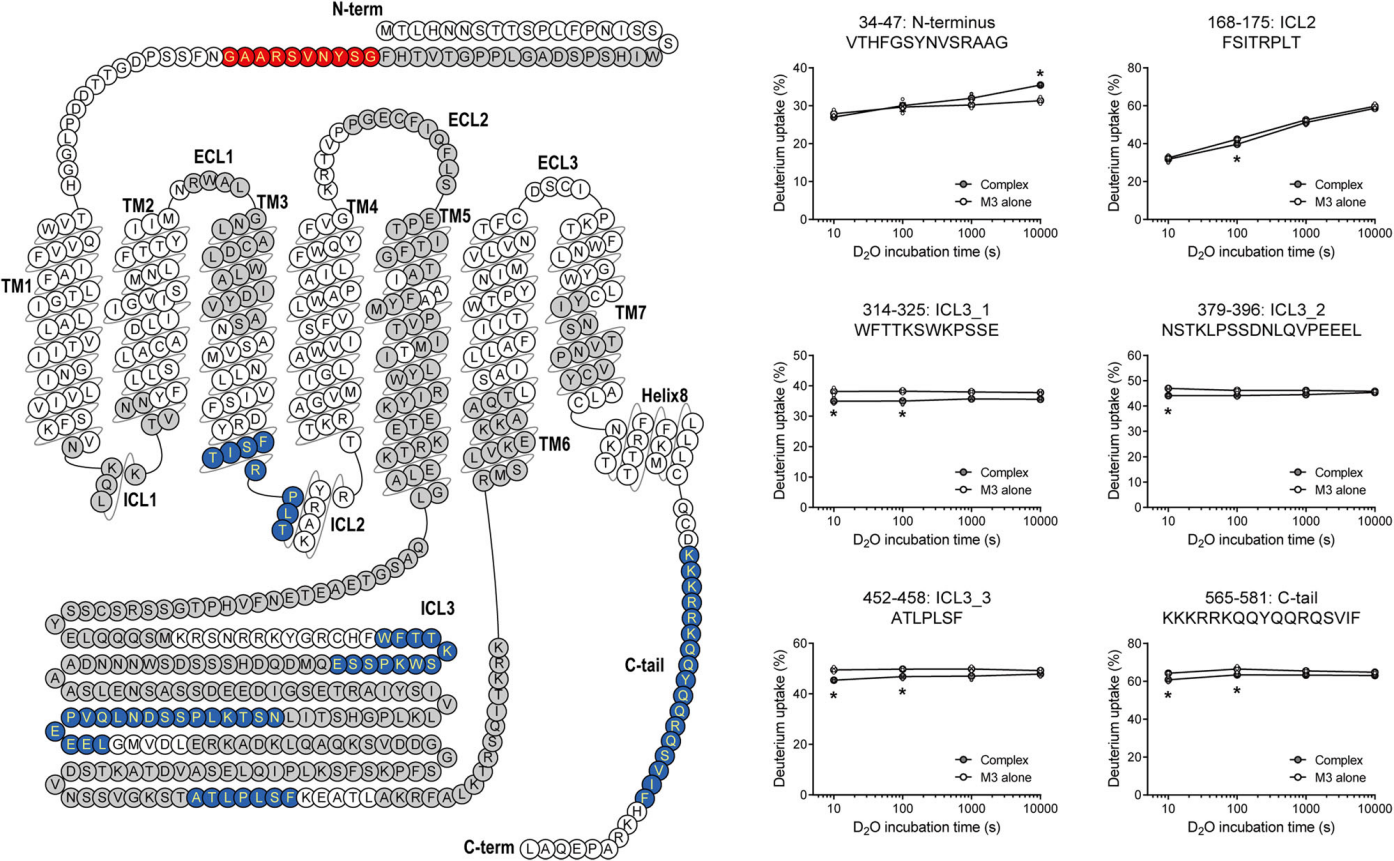
HDX profiles of M3 upon M3-Gq interaction. Changes in the HDX profile of agonist-bound M3 upon co-incubation with heterotrimeric Gq. Regions with reduced and increased HDX levels are colored blue and red, respectively, in the M3 snake plot. The regions in which HDX levels were not altered are shown in gray, and the regions in which peptides were not identified are shown in white. Deuterium uptake plots of selected peptides from the color-coded regions of M3 are shown in the graphs. Results were derived from three independent experiments, and the statistical significance of the differences was determined using Student’s t-test (*p < 0.05). Data are presented as mean ± standard error of the mean. Smaller symbols are individual data points.

To understand the binding interfaces of M3, we analyzed the HDX levels in M3 with or without co-incubation with Gq. M3 showed HDX level changes upon co-incubation with Gq, mostly in the cytosolic regions, including ICL2 (Fig. 4, peptide 168–175), ICL3 (Fig. 4, peptides 314–325, 379–396, and 452–458), and C-tail (Fig. 4, peptide 565–581). Although the HDX level differences were minimal potentially due to weak interaction, HDX levels in these regions were lower in the M3 co-incubated with Gq than in the M3 alone, suggesting that these regions are potential binding interfaces for Gq.

An extracellular region at the M3 N-terminus (Fig. 4, peptide 34–47) showed higher HDX levels in the M3 co-incubated with Gq than in the M3 alone, suggesting that this region underwent allosteric conformational changes upon Gq binding.

### Molecular implication of M3 ICL2 in M3-Gq coupling

Considering that the HDX-MS data suggested that ICL2, ICL3, and the C-tail of M3 are potential binding sites for Gq (Fig. 4), we investigated the functions of these regions in M3-Gq coupling in the cell system using mutant constructs and the NanoBiT assays described in Fig. 3c, d.

Besides the insertion of the Gα C-terminus to the cytosolic core of the receptor, the interaction of the receptor ICL2 with the hydrophobic pocket within Gα formed by the αN/β1 hinge, β2/β3, and α5 has been suggested as critical for receptor-G protein coupling^24,33,34,45,52^. The role of the ICL2 interaction is more profound in GPCR-Gs or GPCR-Gq coupling than in GPCR-Gi coupling^53,54^. Specifically, residue 34.51 of the receptors (residue numbering is based on the GPCRdb numbering scheme^55^), is often a bulky hydrophobic amino acid that forms extensive hydrophobic contact with the Gα hydrophobic pocket in the GPCR-Gs or GPCR-Gq complexes, but not in the GPCR-Gi complexes^11,34,45,52,56^.

**Fig. 5:**
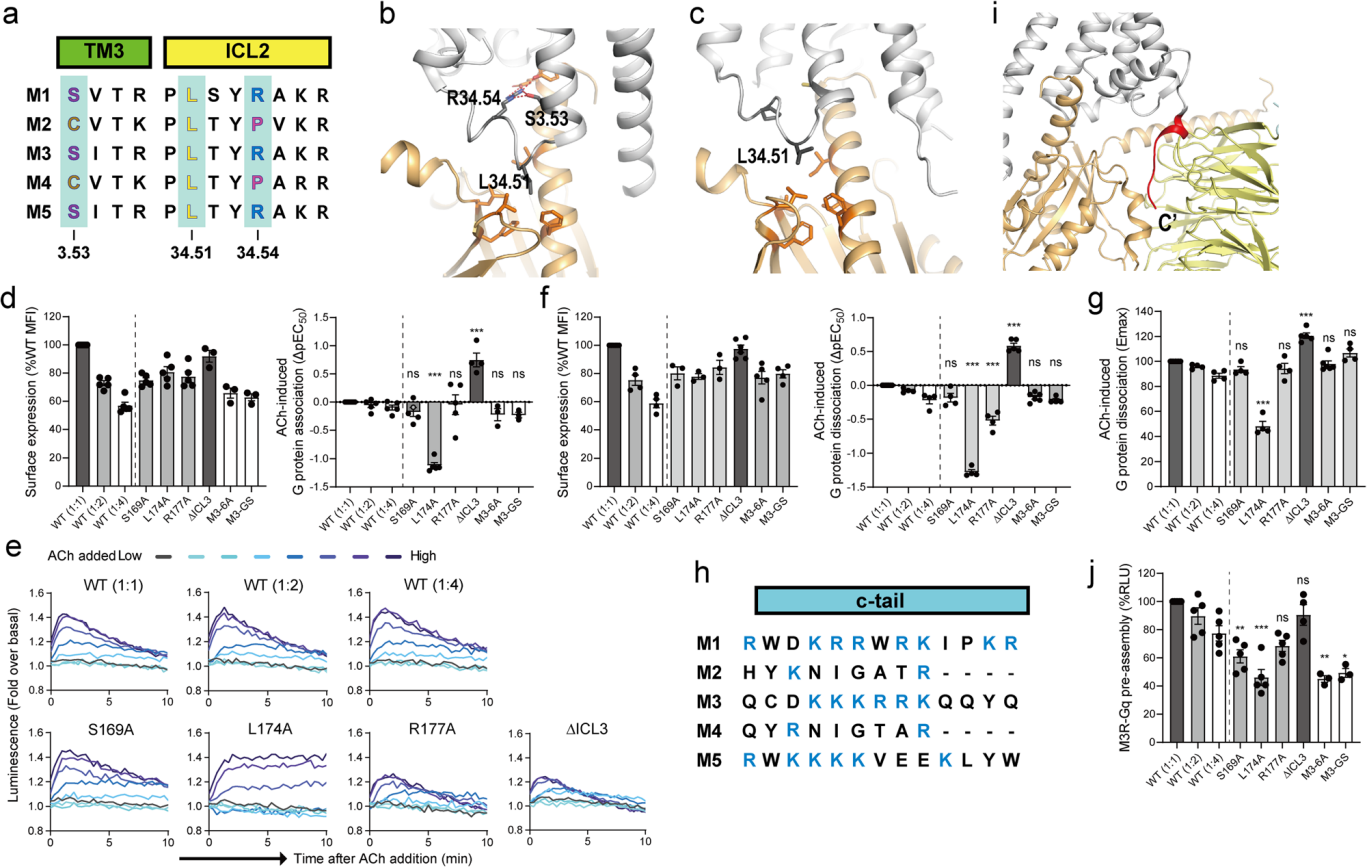
The roles of ICL2, ICL3, and C-tail in M3-Gq coupling. **a** Sequence alignment of muscarinic acetylcholine receptors at the C-terminus of TM3 through ICL2. **b**, **c** Interaction of M3 ICL2 with Gαq (**b**) and M2 ICL2 with GαoA (**c**). Selected residues at the receptor-G protein interface are depicted as sticks on the crystal structures of M3-Gq (PDB: 8E9Z) and M2-GoA (PDB: 6OIK). The receptors are colored grey and Gα is colored light-orange. **d** Measurement of ΔpEC_50_ of ligand-induced M3-Gq interaction using the NanoBiT assay with titrated concentrations of acetylcholine (Ach). The surface expression levels of M3 constructs are shown in the left panel. M3-SmBiT and mutant M3-SmBiT were expressed together with the Gαq–LgBiT, Gβ_1_, and Gγ_2_. WT (1:1), WT (1:2) and WT (1:4) refer to volume of transfected plasmids. Data are from 3-5 independent experiments (dots) with bars and error bars representing mean and SEM, respectively. Statistical analysis was performed by the ordinary one-way ANOVA followed by the Sidak’s post-hoc test with expression-matched (colored) WT conditions. ns, *P* > 0.05; ***P < 0.001. **e** Measurement of M3-Gq interaction kinetics using the NanoBiT assay described in (**d**). Kinetics data are from representative experiments with similar kinetics results. **f** Measurement of ΔpEC_50_ of ligand-induced G-protein dissociation using the NanoBiT assay with titrated concentrations of ACh. The surface expression levels of M3 constructs are shown in the left panel. Gαq–LgBiT, SmBiT–Gγ_2_, and Gβ_1_ were co-expressed with M3 and mutant M3. WT (1:1), WT (1:2), and WT (1:4) refer to volume of transfected plasmids. Data are from 3-5 independent experiments (dots) with bars and error bars representing mean and SEM, respectively. Statistical analysis was performed by the ordinary one-way ANOVA followed by the Sidak’s post-hoc test with expression-matched (colored) WT conditions. ns, *P* > 0.05; **P < 0.01; ***P < 0.001. **g** Measurement of Emax for ligand-induced G-protein dissociation using the NanoBiT assay described in (**f**). **h** Sequence alignment of muscarinic acetylcholine receptors at the C-tail. Positively charged residues are colored blue. **i** Interaction of the polybasic C-tail of M1 with Gα11 and Gβ interface (PDB: 6OIJ). The receptors are colored grey, and Gα is colored light orange. The M1 C-tail is colored red. **j** Basal M3-Gq interaction levels using the NanoBiT assay in the absence of agonist as described in  (**d**). WT M3-SmBiT and the mutant M3-SmBiT constructs were expressed together with the Gαq–LgBiT, Gβ_1_, and Gγ_2_. WT (1:1), WT (1:2), and WT (1:4) refer to volume of transfected plasmids. Data are from 3-5 independent experiments (dots) with bars and error bars representing mean and SEM, respectively. Statistical analysis was performed by the ordinary one-way ANOVA followed by the Sidak’s post-hoc test with expression-matched (colored) WT conditions. ns, *P* > 0.05; *P < 0.05; **P < 0.01; ***P < 0.001.

Our HDX-MS data also suggested that the C-terminal part of TM3 through the ICL2 of M3 is a potential binding interface, because this region showed lower HDX levels in the M3 co-incubated with Gq than in the M3 alone (Fig. 4). Residue 34.51 was identical throughout the five mAChR subtypes (Fig. 5a), yet residue 34.51 of M3 (i.e., L174^34.51^) extensively contacted the hydrophobic pocket in miniGαq (Fig. 5b), whereas this interaction was lost in the M2-Go complex (Fig. 5c). Other residues within this region were also similar among mAChR subtypes (Fig. 5a); however, a few distinct residues showed differences depending on the coupling G proteins. Gq/11-coupled mAChRs (i.e., M1, M3, and M5) have Ser at residue 3.53 (S169^3.53^ in M3) and Arg at residue 34.54 (R177^34.54^ in M3), but Gi/o-coupled mAChRs (i.e., M2 and M4) have Cys at residue 3.53 and Pro at residues 34.54 (Fig. 5a). In the M3-miniGq cryo-EM structure, R177^34.54^ interacts with the C-terminus of Gαq α5 (E355 and Y356) and S169^3.53^ of M3 (Fig. 5b). Therefore, we tested whether these residues played any role in M3-Gq coupling by introducing mutations (i.e., S169^3.53^A, L174^34.51^A, and R177^34.54^A).

Upon acetylcholine treatment, S169^3.53^A showed the M3-Gq interaction similar to that of the expression-matched WT (Fig. 5d, e) and also showed the Gq activation similar to that of the expression-matched WT (Fig. 5f, g), suggesting that this residue plays a dispensable role in M3-Gq coupling. In contrast, R177^34.54^A showed reduced maximum M3-Gq interaction (Fig. 5e) and reduced Gq activation (Fig. 5f), suggesting that R177^34.54^ contributes to both M3-Gq interaction and GDP release.

L174^34.51^A affected on M3-Gq interaction (Fig. 5d, e) and M3-induced Gq activation (Fig. 5f, g). L174^34.51^A showed a maximum M3-Gq interaction similar to that of WT; however, this interaction was sustained (Fig. 5e). The WT and other tested mutants showed transient interactions with a peak luminescent signal upon 1–2 min after ligand addition (Fig. 5e), likely due to GTP incorporation into the GDP-released empty nucleotide-binding pocket and the subsequent dissociation of Gαq from the receptor. However, without GDP release, GTP cannot be incorporated into the G protein, and Gα cannot be activated and dissociate from the receptor and Gβγ. Therefore, the sustained interaction suggests that L174^34.51^A can interact with Gq but fails to release GDP. Consequently, L174^34.51^A exhibited reduced Gq activation (Fig. 5f, g). These results suggest that L174^34.51^ is not critical for the initial interaction between M3 and Gq but is critical for GDP release from Gq.

### Molecular implication of M3 ICL3 and C-tail in M3-Gq coupling

To understand the molecular implication of M3 ICL3 in M3-Gq coupling, we truncated ICL3 from M3 and analyzed the interaction between ICL3-truncated M3 and Gq and the former’s ability to activate Gq (Fig. 5d–g). Notably, ICL3 deletion decreased the maximum M3-Gq interaction (Fig. 5e), but increased the Gq activation (Fig. 5f, g). This suggests that M3 ICL3 helps interact with Gq in a non- or less-functional form.

The C-tail of M3 has multiple positively charged residues, which are also found in other Gq-coupled mAChRs (i.e., M1 and M5) but not in Gi/o-coupled mAChRs (i.e., M2 and M4) (Fig. 5h). In the M3-miniGq cryo-EM structure, the C-tail of M3 is unmodeled^26^. However, in the M1-G11 cryo-EM structure, the C-tail of M1 interacts with the negatively-charged regions at the Gα11 and Gβγ interface (Fig. 5i). In this study’s HDX-MS analysis, the regions between the Gαq and Gβγ interface (i.e., α2 of Gαq, cyan-colored region in Fig. 3a) showed lower HDX levels in the Gq co-incubated with M3 than in the Gq alone (Fig. 3b, peptide 210–226). Therefore, in the M3-Gq complex, the C-tail of M3 may also interact at the Gαq and Gβγ interface. To test whether the M3 C-tail plays a role in coupling with Gq, we generated mutant constructs in which six positively charged residues (KKKRRK) were mutated into either an Ala (AAAAAA; M3-6A) or a Gly-Ser liner (GSGSGS; M3-GS).

The C-tail mutants did not affect M3-induced Gq activation (Fig. 5f, g), suggesting that the positively charged C-tail was not involved in M3-mediated Gq activation, which is consistent with a previous report^57^. However, the positively charged C-tail has been reported to be an essential binding site for M3-Gq pre-assembly, in which M3 forms a complex with Gq prior to agonist binding but cannot activate Gq^57^. Therefore, we measured the basal interaction (i.e., pre-assembled complex formation) between M3 and Gq, and both M3-6A and M3-GS showed weaker basal interactions than in the WT (Fig. 5j). These data confirmed that the M3 C-tail is necessary for M3-Gq pre-assembled complex formation but not for Gq activation. These results also suggest that ICL2, but not ICL3, may be involved in the pre-assembled complex formation (Fig. 5j).

### M3 ICL3 interacts with Gβγ

**Fig. 6:**
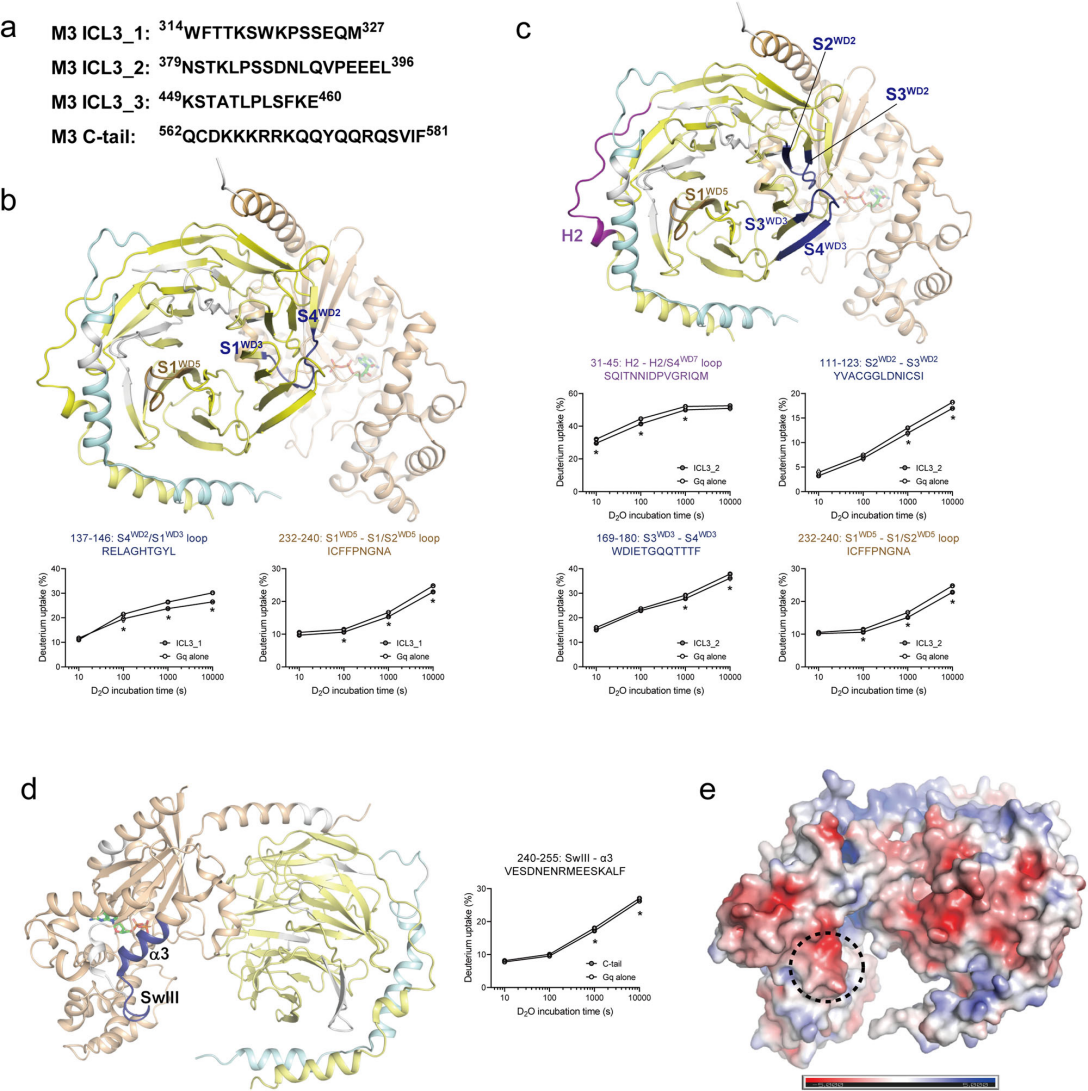
HDX profiles of heterotrimeric Gq upon co-incubation with M3 peptides. **a** Sequences of M3 ICL3 and C-tail peptides. **b**, **c** Changes in the HDX profile of Gβ_1_ upon co-incubation with M3 ICL3_1 (**b**) or M3 ICL_2 (**c**). Regions with altered HDX are color-coded on the structure of inactive heterotrimeric Gq (PDB: 3AH8). The deuterium uptake plots of selected peptides from the color-coded regions are shown as graphs. The regions in which peptides were not identified are shown in white. Results were derived from three independent experiments and the statistical significance of differences was determined using Student’s t-test (*p < 0.05). Data are presented as mean ± standard error of the mean. Smaller symbols are individual data points. **d** Changes in the HDX profile of Gαq upon co-incubation with M3 C-tail peptide. Regions with reduced HDX are colored blue. The deuterium uptake plots of selected peptides with reduced HDX are provided as graphs. The regions in which peptides were not identified are shown in white. Results were derived from three independent experiments and the statistical significance of differences was determined using Student’s t-test (*p < 0.05). Data are presented as mean ± standard error of the mean. Smaller symbols are individual data points. **e** Surface charge distribution of Gq. The surface charge was analyzed using APBS electronics, a PyMOL plugin program, using the heterotrimeric Gq crystal structure (PDB: 3AH8). The Switch III region is shown in the dotted circle.

To identify the specific binding sites of M3 ICL3 on Gq, we generated three peptides from the ICL3 region (Fig. 6a) that showed lower HDX levels in M3 incubated with Gq than in M3 alone (Fig. 4). We analyzed the HDX levels of Gq with or without co-incubation of these peptides (Supplementary Data 3) and observed that the HDX levels of Gαq were not altered by any of these peptides. However, the HDX levels of Gβ_1_ were affected by ICL3_1 and ICL3_2 (Fig. 6b, c) while ICL3_3 did not affect Gβ_1_ HDX levels.

To address potential non-specific interactions, we conducted HDX-MS experiments using peptides designed to abolish binding. For ICL3_1, we generated a scrambled peptide featuring a randomly rearranged sequence compared to that in the WT. In the case of ICL3_2, we introduced alanine mutants, replacing residues with acidic and amide functional groups (D, E, N, and Q) with alanine. We opted not to create a mutant for ICL3_3 as it exhibited no significant effects. We confirmed the absence of alterations in HDX when Gq was co-incubated with mutant ICL3_1 (WTPMEKSSFKTSQW) or mutant ICL3_2 (ASTKLPSSAALAVPEAEL) (Supplementary Data 5), suggesting that the HDX level changes upon co-incubation with ICL3_1 or ICL3_2 occurred because of the specific interaction of these peptides with Gq.

ICL3_1 co-incubation decreased HDX levels in the S4^WD2^/S1^WD3^ loop (peptide 137–146) and S1^WD5^ through the S1/S2^WD5^ loop (peptide 232–240) (Fig. 6b, blue- and brown-colored regions, respectively), suggesting that these two regions interact with ICL3_1. Interestingly, peptide 232–240 also showed lower HDX levels in the M3-Gq complex than in the Gq alone (Fig. 3a, brown-colored region, and Fig. 3e) making the S1^WD5^ through S1/S2^WD5^ loop of Gβ_1_ a strong candidate for the ICL3_1 (residues 314–327) binding region in the M3-Gq complex.

ICL3_2 co-incubation decreased HDX levels in the H2 through H2/S4^WD7^ (peptide 31–45), S2^WD2^ through S3^WD2^ (peptide 111–123), S3^WD3^ through S4^WD3^ (peptide 169–180), and S1^WD5^ through S1/S2^WD5^ loop (peptide 232–240) (Fig. 6c), suggesting that these sites are potential binding sites for the ICL3_2 region. Peptides 111–123 and 169–180 were located in neighboring regions facing opposite sides of Gαq-binding sites (Fig. 6c, blue-colored regions). Interestingly, the peptides 31–45 and 232–240 also showed lower HDX levels in the Gq co-incubated with M3 than in the Gq alone (Fig. 3a, violet- and brown-colored regions, and Fig. 3e, suggesting that the H2 through H2/S4^WD7^ and S1^WD5^ through S1/S2^WD5^ loop of Gβ_1_ are strong candidates for the ICL3_2 (residues 379–396) binding region in the M3-Gq complex.

HDX levels in the blue-colored regions of Fig. 6b, c were altered only when GDP-bound heterotrimeric Gq was co-incubated with ICL3_1 and ICL3_2 peptides, but not when the active nucleotide-free M3-Gq complex was formed (Fig. 3a, e). Notably, these regions may be less accessible in the active and nucleotide-free M3-Gq complex because of the movement of the Gαq AHD. Most of the cryo-EM structures of GPCR-Gq complexes are missing the AHD, but a few currently available GPCR-G protein complex structures show that the position of the Gα AHD varies between structures (Supplementary Fig. 3a). Moreover, a negative-staining EM study suggested that the Gα AHD would exhibit a hanging movement in the nucleotide-free GPCR-G protein complex^58^. The blue-colored regions in Fig. 6b, c are located near the Gα AHD positioned in the nucleotide-free GPCR-G protein complex (Supplementary Fig. 3b) although these blue areas are not as close to the AHD as needed to affect the HDX profiles as seen in previous reports^33–35^. However, although speculative, the hanging movement of the AHD may hinder the access of ICL3, and we suspect that the blue regions in Fig. 6b, c may form contacts with M3 ICL3 only in the M3-Gq pre-assembled complex (i.e., the Gαq AHD is not in the open conformation) but not in the active and nucleotide-free M3-Gq complex (*i.e*., the Gαq AHD is in the open conformation).

Interestingly, the blue-colored regions in Fig. 3a are also near the Gα AHD position (Supplementary Fig. 3c). Thus, we speculate that the blue regions in Fig. 3a showed lower HDX levels in the M3-Gq complex than in the Gq alone due to the movement of the Gαq AHD to Gβγ upon GDP release. However, we cannot rule out the possibility that the blue regions in Fig. 3a interacted with M3 or the detergent micelles.

### M3 C-tail interacts with positively charged regions of Gαq

To identify the specific binding sites of the M3 C-tail on Gq, we generated a peptide from the C-tail (Fig. 6a) that showed lower HDX levels in the M3-Gq complex than in M3 alone (Fig. 4) and analyzed the HDX levels of Gq with or without co-incubation with this peptide (Supplementary Data 4). Co-incubation with the M3 C-tail peptide decreased HDX levels near Switch III (SwIII) of Gαq (Fig. 6d, peptide 240–255). This region is negatively charged (Fig. 6e, circled regions), suggesting that the C-tail peptide might bind to this region through charge-charge interactions. The mass differences of this peptide between the M3-Gq complex and M3 alone were minimal (approximately 0.1 Da) but statistically significant. The small mass differences might be because the charge-charge interactions mediated by the side chains of amino acids did not substantially affect the amide hydrogens in the peptide backbone.

To address potential non-specific interactions, we conducted HDX-MS experiments using a mutant peptide in which basic residues (K and R) were replaced with alanine. We confirmed the absence of any significant difference in HDX when Gq was co-incubated with the mutant C-tail peptide (QCDAAAAAAQQYQQRQSV) (Supplementary Data 5) suggesting that the HDX level changes upon co-incubation with the C-tail peptide occurred because of the specific interaction of this peptide with Gq.

Notably, the Gαq and Gβγ interface, a potential binding site of the C-tail in the M3-Gq complex based on the M1-G11 structure (Fig. 5g), showed lower HDX levels in the Gq co-incubated with M3 than in the Gq alone (Fig. 3a, cyan-colored region, and Fig. 3b, peptide 210–226), but was not affected by the C-tail peptide (Fig. 6d). As the region in Fig. 6d (i.e., SwIII through α3) was altered in the GDP-bound heterotrimeric Gq but not in the nucleotide-free active M3-Gq complex, it is tempting to suggest that the positively charged M3 C-tail interacts at SwIII through α3 in the M3-Gq pre-assembled complex but relocates to the Gαq and Gβγ interface when the active and nucleotide-free M3-Gq complex is formed.

## Discussion

**Fig. 7:**
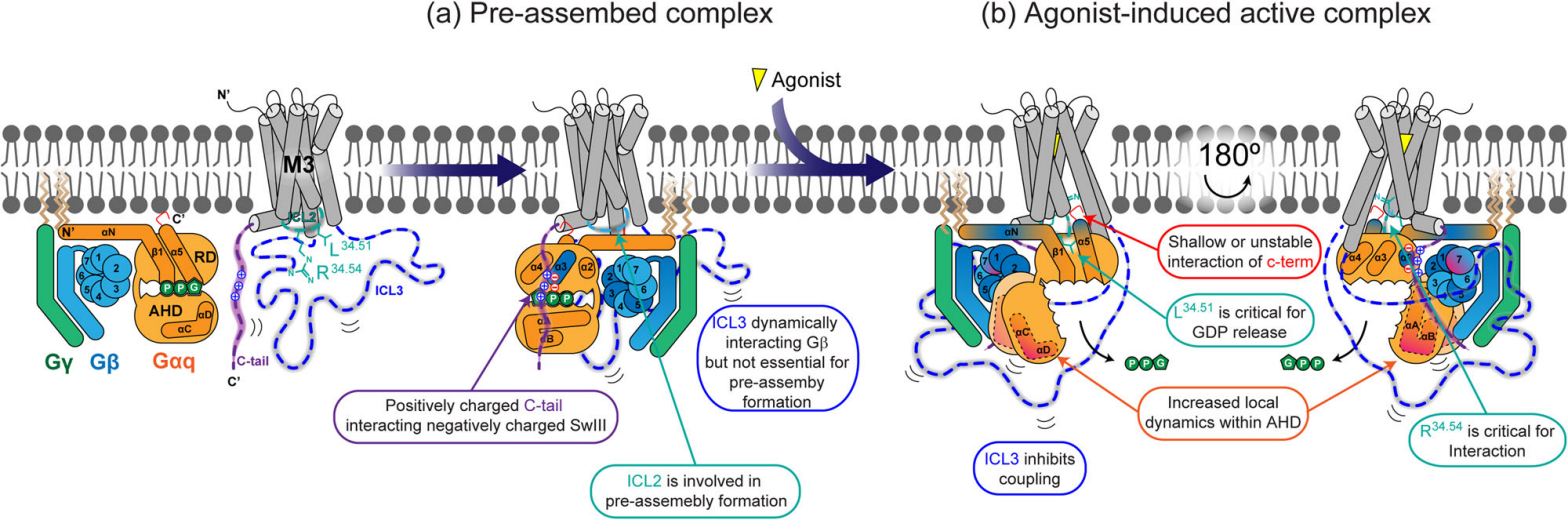
Proposed molecular mechanism of M3-Gq assembly. Schematic illustration of the pre-assembled M3-Gq complex (**a**) and agonist-induced nucleotide-free active-form of M3-Gq complex (**b**). M3 pre-assembles with the heterotrimeric Gq via M3 ICL2 and a positively charged C-tail. Upon agonist binding, M3 is activated and couples to heterotrimeric Gq, resulting in the release of GDP. The potential binding interfaces showing reduced HDX due to complex formation or peptide co-incubation are shown in dark blue. Regions that become more dynamic or unstable in the complex are colored in magenta. R^34.51^ and L^34.54^ are shown as blue and red sticks, respectively.

Unlike previous reports that often used M3 in which ICL3 was truncated or switched to other molecules^12,22,26^, this study used full-length WT M3 and unmodified Gq to understand the coupling mechanism under more physiological conditions. Although HDX-MS was conducted in the detergent micelles with purified proteins, our experimental system using the full-length WT proteins may provide otherwise unelucidated molecular mechanisms. Accordingly, we propose the following molecular mechanism for M3 and Gq coupling (summarized in Fig. 7).

The HDX profile changes of GDP-bound Gq upon co-incubation with C-tail peptide (Fig. 6d) and the relatively low basal interaction between C-tail-mutated M3 and Gq (Fig. 5i) suggest that M3 and Gq may form pre-assembly through the interaction between the positively charged M3 C-tail and negatively charged surface near the SwIII region of Gαq (Fig. 7a). In the pre-assembled M3-Gq complex, the long ICL3 might also interact with Gβγ (Figs. 6b, c, and 7a), but this interaction is not critical for pre-assembled complex formation as we did not observe impaired pre-assembly with the ICL3-truncated M3 construct (Fig. 5i). Moreover, ICL2 may be involved in the pre-assembled complex formation (Fig. 5i) but we could not identify the binding interface. The involvement of the positively charged M3 C-tail in M3-Gq pre-assembled complex formation has been suggested^57^; however, the previous study showed that the positively charged C-tail alone is not sufficient to fully support the pre-assembled complex formation, and it does not define the M3 C-tail binding surface on Gq. Here, we suggest that ICL2 might be the other element for the pre-assembled complex formation and further define the potential binding interfaces in the pre-assembled state: the M3 C-tail interacting with the Gq SwIII region and M3 ICL3 interacting with Gβγ.

Upon agonist binding, M3 was activated and formed an active complex with Gq, resulting in the release of GDP (Fig. 7b). In this active complex, the C-tail of M3 might move to the Gαq and Gβγ interface (Fig. 7b), as we observed HDX levels decrease in this region in the Gq co-incubated with M3 than in the Gq alone (Fig. 3a, b) and the C-tail of M1 is located in this region in the M1-G11 complex (Fig. 5g). In the active M3-Gq complex, the C-tail of M3 is not critical for Gq activation as the C-tail-mutated M3 showed Gq interaction and activation comparable to the WT (Fig. 5d, f).

The C-terminus of Gα α5 has been suggested as critical for GPCR-G protein interaction and selective coupling^33,46,50,59^. Interestingly, the C-terminus of Gαq α5 forms a shallow or relatively unstable interaction with M3 in our experimental system with purified proteins in the detergent micelle (Fig. 7b). This was somewhat unexpected because M3 primarily couples to Gq^60^ and the interaction of Gαq α5 with M3 is extensive in the published cryo-EM structure of the M3-miniGq complex (Fig. 1b)^26^. The shallow or relatively unstable interaction might be more illustrative of the physiological than the cryo-EM structure because the current study used full-length WT M3 and Gq while the cryo-EM structure used ICL3-truncated M3 and chimeric miniGα. Although the interaction might be shallow or relatively unstable, the interaction of the C-terminus of Gαq α5 is still critical for M3-Gq coupling as M3 could not couple to the C-terminus-truncated Gq (Fig. 3c, d).

Another well-defined interface between a GPCR and G protein is the interaction between GPCR ICL2 and the hydrophobic pocket in Gα^11,34,56^. Specifically, we have previously reported that the large hydrophobic residue at position 34.51 of β_2_AR is not responsible for the initial interaction with Gs but is critical for triggering GDP release from Gs^33^. Similar to the β_2_AR-Gs coupling, this study suggests that L174^34.51^ of M3 is not responsible for the initial interaction with Gq (Fig. 5e) but is critical for GDP release from Gq (Fig. 5f). Interestingly, R177^34.54^ of M3, another residue in ICL2, was critical for the early-stage interaction with Gq (Fig. 5e). Thus, we suggest that M3 ICL2 is involved in both early-stage interactions (R177^34.54^) and GDP release (L174^34.51^) (Fig. 7b).

Along with these well-defined interfaces (i.e., the C-terminus of Gαq α5 with M3 and M3 ICL2 with Gαq), this study revealed additional functions of M3 ICL3. As discussed above, M3 has the longest ICL3 among class A GPCRs. We identified two regions within ICL3 that potentially interact with Gβ_1_ of Gq. In the pre-assembled complex (Fig. 7a), these interactions may be extremely dynamic as we observed that the ICL3 peptides affected the HDX profiles of a few different regions in Gβ_1_ (Fig. 6b, c). In the nucleotide-free active M3-Gq complex (Fig. 7b), the AHD movement (Supplementary Fig. 3) may block the binding of ICL3 to a few regions of Gβ_1_; therefore, binding becomes focused on specific regions of Gβ_1_. Interestingly, M3 ICL3 is important for maximum interaction with Gq (Fig. 5e); however, it does not support M3-Gq coupling and instead inhibits it, as we observed that ICL3 truncation increased M3-Gq coupling (Fig. 5f). This is somewhat consistent with a recently published study suggesting that long ICL3s negatively affect receptor-G protein coupling by blocking Gα binding sites^61^. Although M3 ICL3 negatively affects coupling efficiency, it might have positive effects on the colocalization of the signaling components with M3; M3 ICL3 can interact with various downstream molecules such as phospholipase Cβ, calmodulin, and GRK2^25,62^.

This study not only defined the binding interfaces of M3 and Gq but also suggested the allosteric conformational changes within the Gαq AHD (Fig. 7b). M3 binding affected local regions within the Gαq AHD located distantly from the nucleotide-binding pocket (Fig. 2), potentially unwinding the α-helices in these local regions. The conformational changes or functional implications of the Gα AHD have not been extensively studied^63^; however, recently, we reported that the Gαs AHD can act as binding sites for other cytosolic proteins^36^. Therefore, if any Gαq AHD-binding proteins exist, local conformational changes in the Gαq AHD might affect these interactions. Further studies are required to understand the functional implications of the Gαq AHD local conformational changes.

In summary, we identified the binding interfaces between M3 and Gq, including previously well-known and unknown interfaces, in both the pre-assembled and active complexes. We also defined the roles of these binding interfaces in the M3-Gq coupling. However, this study had limitations. First, we cannot exclude the possibility that there are other factors such as membrane lipids that can help to stabilize the receptor-G protein complex formation; for example, the β_2_AR-Gi complex formation is affected by the local membrane charge states^64^. Second, we used the peptides from ICL3 or C-tail of M3 to define the binding interfaces to Gq. As the peptides can form different three-dimensional structures compared to the structures within the protein, the HDX-MS data with the peptides should be interpreted with caution. Therefore, further studies are required to define more physiological condition for the active M3-Gq complex formation and analyze the high-resolution structures in this condition.

## Materials and methods

### Protein expression and purification

WT human Gαq, human His6-Gβ_1_, and bovine Gγ_2_ were cloned into the pVL1392 vector, and Ric8A was cloned into the pFastBac1 vector. G proteins were co-expressed in *Trichoplusia ni* insect cells grown in ESF 921 medium (Expression Systems, Davis, CA, USA). Cell cultures were grown at 27 °C to a density of 3 × 10^6^ cells/mL and then infected with Gαq, Gβ_1_γ_2_, and Ric8A baculovirus (40 mL/L, 20 mL/L, and 10 mL/L respectively). After 48 h of incubation, the infected cells were harvested by centrifugation and stored at −80 °C until use. Cell pellets were resuspended in 150 mL lysis buffer (10 mM Tris, pH 7.5, 0.1 mM MgCl_2_, 5 mM β-mercaptoethanol [β-ME], 10 μM GDP, 2.5 μg/mL leupeptin, and 160 μg/mL benzamidine) per liter of culture volume and stirred at room temperature for 15 min. Cell membranes were then centrifuged and resuspended in 100 mL solubilization buffer (20 mM HEPES, pH 7.5, 100 mM NaCl, 1% sodium cholate, 0.05% dodeclymaltoside [DDM], 5 mM MgCl_2_, 2 μL calf intestinal alkaline phosphatase [CIP], 5 mM β-ME, 10 μM GDP, 2.5 μg/mL leupeptin, and 160 μg/mL benzamidine) per liter of culture volume using a 40 mL Dounce homogenizer and tight pestle. The samples were stirred at 4 °C for 1 h and then centrifuged for 20 min to remove insoluble debris. Nickel-IDA resin (2 mL/L cell culture) pre-equilibrated in solubilization buffer was added to the supernatant and shaken for 2 h at 4 °C. After incubation, the Ni-IDA resin was centrifuged, poured into a glass column, and washed with 50 mL solubilization buffer. The heterotrimeric Gq was then gradually exchanged into E2 buffer (20 mM HEPES, pH 7.5, 50 mM NaCl, 0.1% DDM, 1 mM MgCl_2_, 5 mM β-ME, 10 μM GDP, 2.5 μg/mL leupeptin, and 160 μg/mL benzamidine). Proteins were eluted using E2 buffer supplemented with 250 mM imidazole. Proteins were then dephosphorylated by treatment with 5 μL lambda phosphatase (supplemented with 1 mM MnCl_2_ for activity), 1 μL CIP, and 1 μL antartic phosphatase, and incubated at 4 °C overnight. Proteins were further purified using a HiTrap Q HP 5 mL column (Cytiva, Uppsala, Sweden). Peak fractions of the HiTrap Q column were collected and concentrated using a Millipore concentrator with a molecular weight cut-off of 100 kDa. The concentrated heterotrimeric Gq was aliquoted and frozen at −80 °C before use.

Human M3 genes containing N-terminal FLAG tags and C-terminal His-tags were subcloned into the pFastBac1 vector. M3 was expressed in Sf9 insect cells (Expression Systems, 94-001 F) using the Bac-to-Bac baculovirus system. Sf9 cells were grown in ESF 921 medium and infected with the recombinant baculovirus at a density of 4 × 10^6^ cells/mL in the presence of 10 μM atropine. The cells were harvested after 48 h after infection at 27 °C. Cell pellets were lysed using a lysis buffer (10 mM Tris, pH 7.5, 1 mM EDTA, 10 μM atropine, 2.5 μg/mL leupeptin, and 160 μg/mL benzamidine). Subsequently, cell membranes were centrifuged and solubilized with a buffer containing 20 mM HEPES pH 7.5, 750 mM NaCl, 1% DDM, 0.2% sodium cholate, 0.03% cholesterol hemisuccinate (CHS), 10 μM atropine, 2.5 μg/mL leupeptin, 160 μg/mL benzamidine, and 30% glycerol. The solubilized receptor was then purified by Ni-NTA chromatography and eluted with a pH 7.5 buffer containing 20 mM HEPES, 750 mM NaCl, 0.1% DDM, 0.02% sodium cholate, 0.03% CHS, 10 μM atropine, and 30% glycerol, and supplemented with 250 mM imidazole. The Ni-NTA-purified receptor was then loaded onto an anti-FLAG column with M1 affinity resin and washed extensively with a pH 7.5 buffer containing 20 mM HEPES, 750 mM NaCl, 0.1% DDM, 0.02% sodium cholate, 0.003% CHS, and 10 μM iperoxo, and supplemented with 2 mM CaCl_2_. Subsequently, the receptor was eluted with the same buffer supplemented with 0.2 mg/mL FLAG peptide and 5 mM EDTA. The anti-FLAG-chromatography-purified receptor was purified using size-exclusion chromatography against a pH 7.5 buffer containing 20 mM HEPES, 100 mM NaCl, 0.1% DDM, 0.003% CHS, and 10 μM iperoxo. The monodisperse peak fractions were concentrated, flash frozen, and stored at −80 °C until further use.

### Peptide synthesis

Peptide synthesis was performed by Peptron Inc. (Daejeon, Republic of Korea). Briefly, the peptide was synthesized by Fmoc solid phase peptide synthesis (SPPS) using ASP48S (Peptron Inc., Daejeon, Korea) and purified by reverse phase HPLC using a capcell pak C18, 5 μm, 120 Å column (4.6 × 50 mm; Shiseido, Tokyo, Japan). The molecular weight of the purified peptide was confirmed using liquid chromatography-mass spectrometry (LCMS-2020; Shimadzu, Kyoto, Japan).

### Co-incubation protocol

To form the M3-Gq complex, iperoxo-bound M3 and heterotrimeric Gq protein were mixed at a final concentration of 50 μM at room temperature for 4 h. Apyrase (200 mU/mL) was added after 90 min of incubation to hydrolyze GDP and to generate a stable complex. For the complex formation of Gq with M3 peptides, heterotrimeric Gq was mixed with each peptide (500 μM) at a 10-fold molar excess relative to heterotrimeric Gq (50 μM) at room temperature for 1 h.

### HDX-MS

For the M3-Gq complex, hydrogen/deuterium exchange was initiated by mixing 5 μL of protein sample and 25 μL of D_2_O buffer (20 mM HEPES, pD 7.4, 100 mM NaCl, 0.1% DDM, 1 mM MgCl_2_, and 100 μM tris(2-carboxyethyl) phosphine hydrochloride (TCEP) supplemented with 5 μM iperoxo or 10 μM GDP for the complex or alone samples, respectively) and incubated for 10, 100, 1,000, and 10,000 s at room temperature. The deuterated samples were quenched using 30 μL of ice-cold quench buffer (60 mM NaH_2_PO_4_, pH 2.01, 20 mM TCEP, and 10% glycerol), snap-frozen on dry ice, and stored at −80 °C. Non-deuterated (ND) samples were prepared by mixing 5 μL of the protein sample with 25 μL of their respective H_2_O buffers, followed by quenching and freezing, as described above. For M3 peptides-Gq complex, 5 μL of protein sample and 25 μL of D_2_O buffer (20 mM HEPES, pD 7.4, 100 mM NaCl, 0.1% DDM, 1 mM MgCl_2_, 100 μM TCEP, and 10 μM GDP) were mixed, incubated, and quenched as described above.

The quenched samples were digested by passing through an immobilized pepsin column (2.1 × 30 mm; Life Technologies, Carlsbad, CA, USA) at a flow rate of 100 µL/min with 0.05% formic acid in H_2_O at 12 °C. The peptide fragments were subsequently collected on a C18 VanGuard trap column (1.7 µm × 30 mm; Waters, Milford, MA, USA) and desalted with 0.05% formic acid in H_2_O. Peptic peptides were then separated using ultra-pressure liquid chromatography (UPLC) on an ACQUITY UPLC C18 column (1.7 µm, 1.0 mm × 100 mm; Waters) at 40 µL/min with an acetonitrile gradient created by two pumps—mobile phase A (0.15% formic acid in H_2_O) and B (0.15% formic acid in acetonitrile). The gradient started at 8% B and increased to 85% B over 8.5 min. To minimize the back-exchange of deuterium to hydrogen, the sample, solvents, trap, and UPLC column were all maintained at pH 2.5 and 0.5 °C during analysis. Mass spectrometry analyses were performed using a Xevo G2 QTof (Waters) or Xevo G2-XS Qtof (Waters) equipped with a standard electrospray ionization (ESI) source in the MS^E^ mode (Waters) in positive ion mode. The capillary, cone, and extraction cone voltages were set to 3 kV, 40 V, and 4 V, respectively. The source and desolvation temperatures were set to 120 °C and 350 °C, respectively. The trap and transfer collision energies were set to 6 V and the trap gas flow was set to 0.3 mL/min. The mass spectrometer was calibrated with sodium iodide solution (2 µg/µL). [Glu1]-Fibrinopeptide B solution (200 fg/µL) in MeOH:water (50:50 [v/v] + 1% acetic acid) was utilized for the lock-mass correction, and the ions at mass-to-charge ratio (m/z) 785.8427 were monitored at a scan time of 0.1 s with a mass window of ± 0.5 Da. The reference internal calibrant was introduced at a flow rate of 20 µL/min using a lock mass sprayer, and the acquired spectra were automatically corrected using the lock mass. Subsequently, two independent interleaved acquisitions were automatically created: the first function, typically set at 4 eV, collected low-energy or unfragmented data, whereas the second function collected high-energy or fragmented data, typically obtained using a collision ramp from 30 to 55 eV. Argon (Ar) gas was used for collision-induced dissociation (CID). Mass spectra were acquired in the m/z range of 100–2000 for 10 min. Peptides were identified in the ND samples using the ProteinLynx Global Server 2.4 (Waters). The following parameters were applied: monoisotopic mass, nonspecific for the enzyme while allowing up to one missed cleavage, MS/MS ion searches, automatic fragment mass tolerance, and automatic peptide mass tolerance. Searches were performed with variable methionine oxidation modifications, and the peptides were filtered with a peptide score of six. To process the HDX-MS data, the amount of deuterium in each peptide was determined by measuring the centroid of the isotopic distribution using DynamX 3.0 (Waters). Subsequently, the EX1 kinetics were determined by visually inspecting the shape of the isotopic distribution of all peptides. The detailed HDX-MS data of all analyzed peptides are summarized in Supplementary Data.

### Plasmid construction

For the NanoBiT-G protein dissociation assay, full-length human M3 was inserted into the pCAGGS expression vector with an N-terminal fusion of the hemagglutinin-derived signal sequence (ssHA), a FLAG epitope tag, and a flexible linker (MKTIIALSYIFCLVFADYKDDDDKGGSGGGGSGGSSSGGG; the FLAG epitope tag is underlined). The resulting construct was named ssHA-FLAG-M3. For the NanoBiT-G-protein coupling assay, ssHA-FLAG-M3 was C-terminally fused with a flexible linker and the SmBiT fragment GGSGGGGSGGSSSGGVTGYRLFEEIL (SmBiT is underlined). The resulting construct was named ssHA-FLAG-M3-SmBiT.

### NanoBiT G protein dissociation assay

Ligand-induced Gq dissociation was measured using a NanoBiT-G-protein dissociation assay, in which the interaction between a Gα subunit and a Gβγ subunit was monitored by the NanoBiT system (Promega, Madison, WI, USA). Specifically, a NanoBiT-Gq protein consisting of a Gαq subunit fused with a large fragment (LgBiT) at the AHD (between residues 97 and 98 of Gαq) and an N-terminally small fragment (SmBiT)-fused Gγ_2_ subunit was expressed along with an untagged Gβ_1_ subunit and ssHA-FLAG-M3. HEK293A cells were seeded in a six-well culture plate at a concentration of 2 × 10^5^ cells/mL (2 mL per well in DMEM [Nissui, Tokyo, Japan] supplemented with 10% fetal bovine serum [Gibco, Thermo Fisher Scientific, Waltham, MA, USA], glutamine, penicillin, and streptomycin) one day before transfection. The transfection solution was prepared by combining 5 µL (per dish hereafter) polyethylenimine (PEI) Max solution (1 mg/mL; Polysciences, Niles, IL, USA), 200 µL Opti-MEM (Thermo Fisher Scientific), and a plasmid mixture consisting of 200 ng ssHA-FLAG-M3 (or an empty plasmid for mock transfection), 100 ng LgBiT-containing Gαq subunit, 500 ng Gβ_1_ subunit, 500 ng SmBiT-fused Gγ_2_ subunit, and 100 ng RIC8A. After incubation for 1 day, the transfected cells were harvested using 0.5 mM EDTA-containing Dulbecco’s PBS (D-PBS), centrifuged, and suspended in 2 mL Hank’s Balanced Salt Solution (HBSS) containing 0.01% bovine serum albumin (BSA; fatty acid-free grade; SERVA, Heidelber, Germany) and 5 mM HEPES (pH 7.4) (assay buffer). The cell suspension was dispensed into a white 96-well plate at a volume of 80 µL per well and loaded with 20 µL of 50 µM coelenterazine (Angene, London, England) diluted in the assay buffer. After 2 h incubation at room temperature, the plate was measured for baseline luminescence (SpectraMax L; Molecular Devices, San Jose, CA, USA) and titrated concentrations of acetylcholine (20 µL; 6X of final concentrations) were manually added. The plate was immediately read for the second measurement in the kinetic mode, and the luminescence counts recorded from 5 to 10 min after compound addition were averaged and normalized to the initial counts. The fold-change values were normalized to those of the vesicle-treated samples and used to plot the G protein dissociation response. Using Prism 9 software (GraphPad Prism, Boston, MA, USA), the G protein dissociation signals were fitted to a four-parameter sigmoidal concentration-response curve with a *HillSlope* constraint of absolute values less than 2. For each replicate experiment, the parameters *Span* (*Top* – *Bottom*) and pEC_50_ (negative logarithmic values of EC_50_ values) of the individual M3 mutants were normalized to those of the WT M3 performed in parallel, and the resulting *E*_*max*_ values were used to calculate the ligand response activity of the mutants.

### NanoBiT G protein association assay

Ligand-induced Gq coupling was performed as previously described with minor modifications. Transfection was performed according to the same procedure described above, except a plasmid mixture consisting of 500 ng ssHA-FLAG-M3-SmBiT, 500 ng LgBiT-containing Gα_q_, 500 ng Gβ_1_, 500 ng Gγ_2_, and 100 ng RIC8A (G-protein chaperon) was used. The transfected cells were dispensed into a 96-well plate and ligand-induced luminescent changes were measured using the same procedures as those described for the NanoBiT-G-protein dissociation assay.

### Flow cytometry

Transfection was performed according to the same procedure described in the above sections. Subsequently, one day after transfection, the cells were collected by adding 200 μL of 0.53 mM D-PBS, followed by 200 μL of 5 mM HEPES (pH 7.4)-containing HBSS. The cell suspension was transferred to a 96-well V-bottom plate in duplicate and fluorescently labeled with an anti-FLAG epitope (DYKDDDDK) tag monoclonal antibody (Clone 1E6, FujiFilm Wako Pure Chemicals, Osaka, Japan; 10 μg/mL diluted in 2% goat serum- and 2 mM EDTA-containing D-PBS [blocking buffer]) and a goat anti-mouse IgG secondary antibody conjugated with Alexa Fluor 488 (Thermo Fisher Scientific, 10 μg/mL diluted in the blocking buffer). After washing with D-PBS, the cells were resuspended in 200 μL of 2 mM EDTA-containing-D-PBS and filtered through a 40 μm filter. The fluorescence intensity of single cells was quantified using an EC800 flow cytometer (Sony, Tokyo, Japan) equipped with a 488 nm laser (Sony). The fluorescent signal derived from Alexa Fluor 488 was recorded using an FL1 channel, and flow cytometry data were analyzed using the FlowJo software (FlowJo, Ashland, OR, USA). Live cells were gated with a forward scatter (FS-peak-lin) cutoff of 390, with a gain of 1.7. Mean fluorescence intensity (MFI) values of approximately 20,000 cells per sample were used for the analysis. Typically, we obtained MFI values of 2700 (arbitrary units) for ssHA-FLAG-M3, 1800 for ssHA-FLAG-M3-SmBiT, and 20 for mock transfection. For each experiment, we normalized the MFI value of the mutants to that of the WT performed in parallel, and denoted the relative levels.

### BODIPY-FL-GTPγS assay

Nucleotide-binding to heterotrimeric Gq was determined by measuring changes in the fluorescence intensity of BODIPY-FL-GTPγS (ThermoFisher Scientific, Waltham, MA, USA) in an imaging buffer comprised of 20 mM Tris-HCl (pH 8.0), 1 mM EDTA, 10 mM MgCl_2_, and 100 μM TCEP. The fluorophore was excited at 485 nm (bandwidth 14 nm) and the emission spectrum was recorded at 535 nm (bandwidth 25 nm) using TriStar2 S LB 942 Multimode Microplate Reader (Berthold, Germany). Heterotrimeric Gq and M3 prepared in 20 mM HEPES (pH 7.4), 100 mM NaCl, 0.1% DDM, 5 μM iperoxo, 1 mM MgCl_2_, 100 μM TCEP, and 10 μM GDP were mixed with imaging buffer with or without 250 nM BODIPY-FL-GTPγS in 1:10 dilution (1.5 μM final M3 and heterotrimeric Gq concentration). The baseline values in the absence of protein samples was determined by measuring the fluorescence intensity of the imaging buffer with or without 250 nM BODIPY-FL-GTPγS for 120 s. Then, M3 and heterotrimeric Gq were added in order and mixed rapidly in the fluorescence well. The changes in fluorescence were measured for 20 min. Data points were collected every 10 s using a black 96-well plate. All steps were carried out at room temperature. The spectra were corrected by measurements taken in the absence of BODIPY-FL-GTPγS. The resulting kinetics spectra were plotted using GraphPad Prism 8.0.

### Statistics and reproducibility

For HDX-MS analysis, a Student’s *t*-test was used to assess the statistically significant differences between samples with and without the binding partner. A one-way analysis of variance (ANOVA) followed by Tukey’s post-hoc test was used to analyze the differences between more than three conditions. Statistical significance was set at *p* < 0.05. More than three independent experiments were performed for each dataset.

### Reporting summary

Further information on research design is available in the Nature Portfolio Reporting Summary linked to this article.

### Supplementary information

Supplementary Information

Description of additional supplementary files

Supplementary Data

Reporting Summary

## Data Availability

All the data supporting the findings of this study are included in the manuscript and its Supplementary Information files. HDX-MS data have been deposited to ProteomeXchange Consortium^65^ via PRIDE^66^ partner repository with the set identifier PXD042562. The source data underlying Figs. 1c, 2, 3b, e, 4, and 6b–d are provided as a Source Data file.
