# Supplementary material for: Molecular mechanism of muscarinic acetylcholine receptor M3 interaction with Gq
Source: Commun Biol. 2024 Mar 23;7:362. doi: 10.1038/s42003-024-06056-1 (PMC10960872; doi:10.1038/s42003-024-06056-1)
Supplement: Supplementary file 1 — Supplementary Information [file 42003_2024_6056_MOESM1_ESM.pdf]

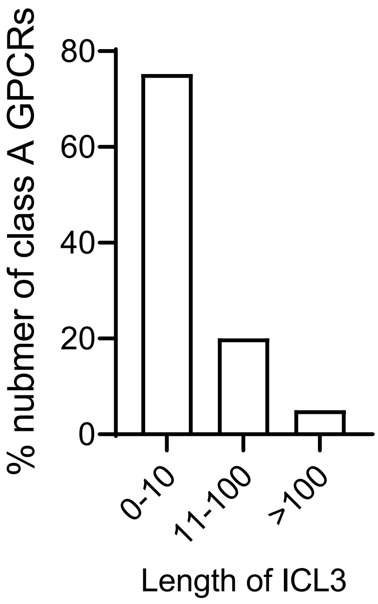

Supplementary Fig. 1. Length of the intracellular loop 3 of class A GPCRs excluding odorant receptors.

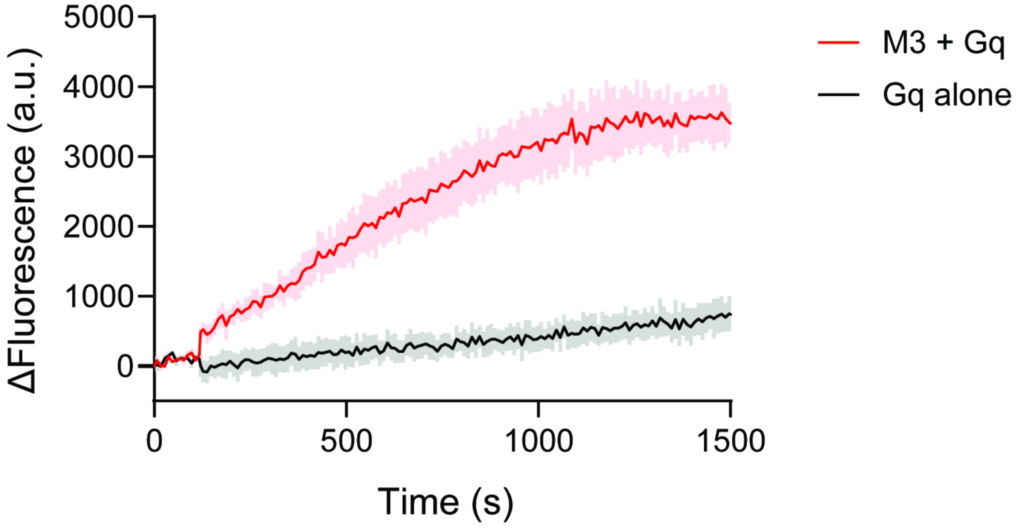

Supplementary Fig. 2. Functional coupling between M3 and Gq.
GDP/GTP turnover was monitored using BODIPY-FL-GTPγS. Fluorescence of 250 nM BODIPY-FL-GTPS was measured to establish the baseline, and then 1.5 μM Gq with or without 1.5 μM M3 was added after 120 s. The curves (red for M3+Gq and gray for Gq alone) represent the mean ± S.E.M of three independent experiments.

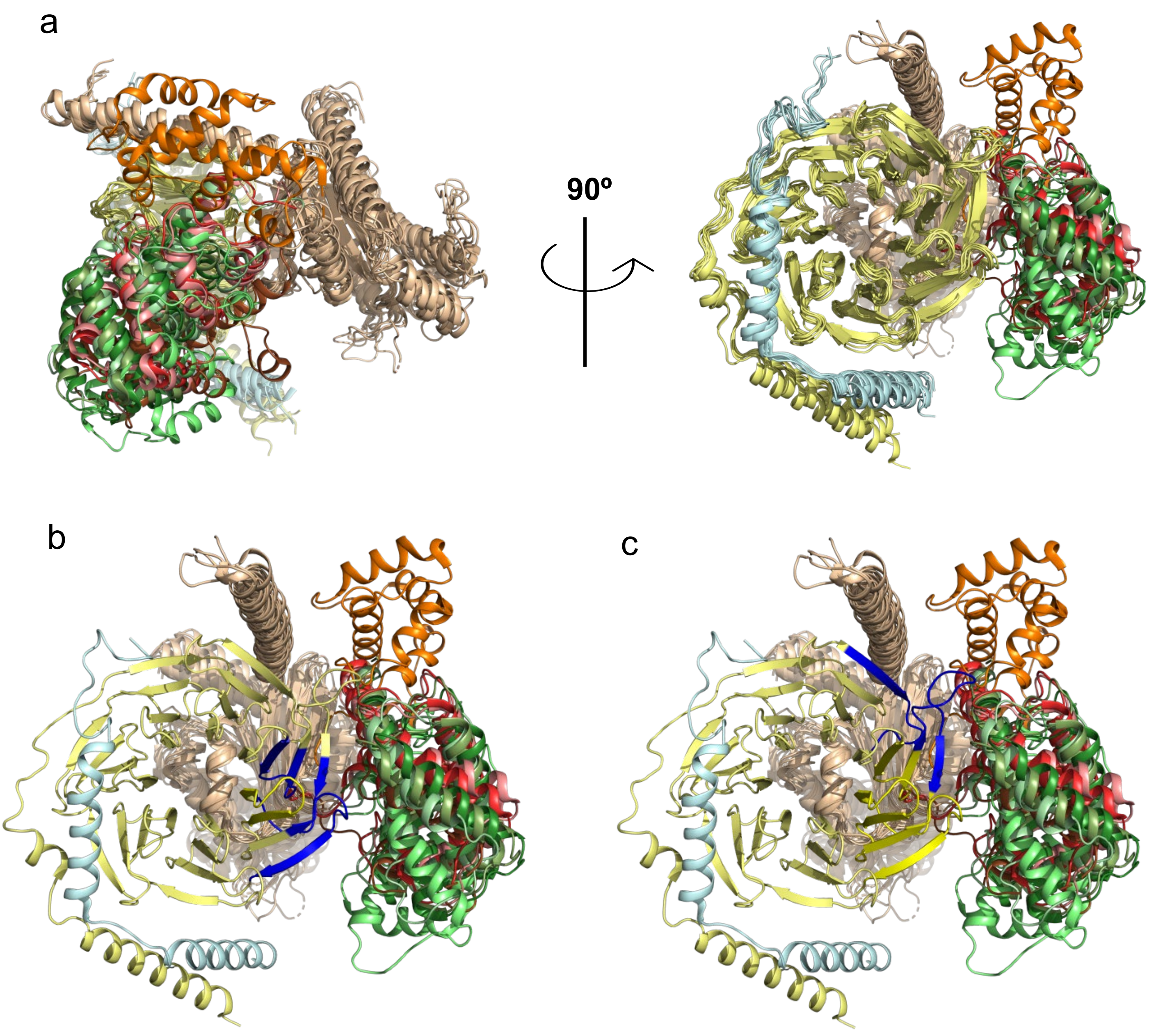

Supplementary Fig. 3. The α-helical domains of Gα contact Gβγ.

(a) Superposition of class A GPCR-G protein complex structures with resolved AHD (Cannabinoid receptor CB2-Gi1, PDB: 6PT0; Rhodopsin-Gi1, PDB: 6CMO; NTS1-Gi1, PDB: 7L0Q; neurotensin receptor 1 NTS1-Gi1 in NC state, PDB: 7L0S; luteinizing hormone–choriogonadotropin receptor LHCGR(S277I)-Gs, PDB: 7FIG; LHCGR(WT)-Gs, PDB: 7FII; β_2AR-Gs, PDB: 3SN6; dopamine receptor D1-Gs, PDB: 7JOZ). Ras-like domain (RD) of Gα is colored in light orange, α-helical domain (AHD) of Gα is in various color. Gβ is colored in yellow and Gγ is colored in cyan. Receptors are not shown.

(b) Regions colored in blue are the regions color-coded blue in Figs. 6b and 6c.

(c) Regions colored in blue are the regions color-coded blue in Figs. 3a and 3e.

Supplementary Table 1. Receptor or Gαq engineering and auxiliary proteins in the published GPCR-Gq structure

PDB ID	Complex	Receptor Engineering	Gαq Engineering	Auxiliary Proteins
6WHA	$5HT_{2A}$-Gq	ΔN65, ΔC405*, ICL3 deleted, BRIL (ICL3)	mini-Gq-Gi2-Gs chimera	scFv16
7DFL	H1-Gq	ΔN27, ΔC488, ICL3 deleted, LgBiT (C-term)	Gqi1N	scFv16
7EIB	B1-Gq	BRIL (N-term), LgBiT (C-term)	mini-Gq-Gi1-Gs chimera	-
7EZM	CCK1-Gq	WT**	Gqi1N	scFv16
7F2O	B2-Gq	ΔN39, ΔC371, BRIL (N-term), LgBiT (C-term)	mini-Gq-Gi1-Gs chimera	scFv16
7F8W	CCK2-Gq	ΔC419	Gqi1N	scFv16
7F9Y	Ghrelin-Gq	BRIL (N-term), LgBiT (C-term)	mini-Gq-Gi1-Gs chimera	scFv16 Nanobody-35
7MBY	CCK1-Gq	WT	mini-Gq-Gi1-Gs chimera	-
7P00	NK1-Gq	ΔC336	mini-Gq-Gi1-Gs chimera	scFv16
7RYC	OT-Gq	WT	mini-Gq-Gi2-Gs chimera	scFv16
7S8L	MRGPRX2-Gq	BRIL (N-term)	mini-Gq-Gi2-Gs chimera	scFv16
7WQ4	GAL2-Gq	LgBiT (C-term)	mini-Gq-Gi1-Gs chimera	Nanobody-35
8E9Z	M3-Gq	ICL3 deleted, BRIL (N-term), LgBiT (C-term)	mini-Gq-Gi2-Gs chimera	scFv16
8HCX	ETBR-Gq	ΔN26, ΔC425, LgBiT (C-term)	mini-Gq-Gi2-Gs chimera	scFv16

*ΔN means truncation of N-terminal residues, ΔC means truncation of C-terminal residues,
**WT refers to wild type, full-length construct.
Abbreviations: $5HT_{2A}$, 5-hydroxytryptamine receptor 2A; H1, Histamine H1 receptor; B1, B1 bradykinin receptor; CCK1, Cholecystokinin receptor type A; B2, B2 bradykinin receptor; CCK2, Gastrin/cholecystokinin type B receptor; Ghrelin, Growth hormone secretagogue receptor type 1; NK1, Substance-P receptor; OT, Oxytocin receptor; MRGGPRX2, Mas-related G-protein coupled receptor member X2; GAL2, Galanin receptor type 2; M3, Muscarinic acetylcholine receptor M3; ETBR, Endothelin receptor type B; BRIL, thermostabilized apocytochrome b_{562}RIL; LgBiT, large complementation fragment; scFv16, single-chain variable fragment 16; PDB, protein Data Bank
