# Supplementary material for: Molecular mechanism of muscarinic acetylcholine receptor M3 interaction with Gq
Source: Commun Biol. 2024 Mar 23;7:362. doi: 10.1038/s42003-024-06056-1 (PMC10960872; doi:10.1038/s42003-024-06056-1)
Supplement: Supplementary file 2 — Description of additional supplementary files [file 42003_2024_6056_MOESM2_ESM.pdf]

Description of Additional Supplementary Files

File name: Supplementary Data 1

Description: Summary of continuous labeling HDX-MS data (Figure 1-4, 6, and Supplementary Data 2-6).

File name: Supplementary Data 2

Description: Deuterium uptake levels of HDX-MS of M3-Gq (Figure 1-4).

File name: Supplementary Data 3

Description: Deuterium uptake levels of HDX-MS of M3 ICL3 peptides-Gq (Figure 6b-c).

File name: Supplementary Data 4

Description: Deuterium uptake levels of HDX-MS of M3 C-tail peptide-Gq (Figure 6d).

File name: Supplementary Data 5

Description: Deuterium uptake levels of HDX-MS of M3 mutant peptides-Gq.

File name: Supplementary Data 6

Description: Deuterium uptake levels of HDX-MS of Gq upon co-incubation with apyrase.
